# Detailed visualization and morphometric analysis of reconstructed neurons using Blender and Python

**DOI:** 10.1186/1471-2202-12-S1-P323

**Published:** 2011-07-18

**Authors:** Paulo Aguiar, Peter Szucs

**Affiliations:** 1 Centro de Matematica da Universidade do Porto, Portugal; 2 Spinal Neuronal Networks, Instituto de Biologia Molecular e Celular, Portugal

## 

Topology and functional features are two related aspects in a neuron. Understanding and measuring the neuron's topology is therefore an important step in inferring and constraining its functional properties. Unfortunately the sheer complexity of most neuron's structures makes it virtually impossible to describe the topology richness in just a handful of parameters. Ultimately, the best way to describe a neuron's topology is by plainly performing reconstruction and visualizing it in a virtual 3D space. Here we present a collection of scripts, written in python programming language, which use Blender for 3D visualization. Blender is a well established free open source 3D content creation suite, available for all major operating systems under the GNU General Public

**Figure 1:**
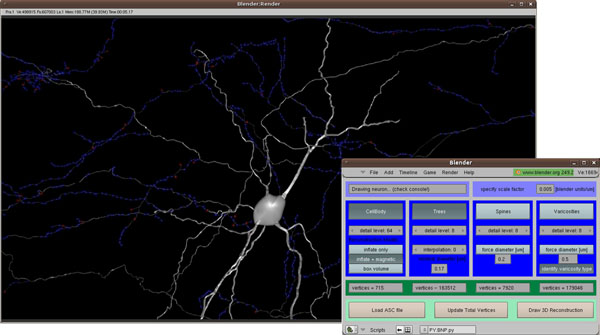
Rendered neuron using the visualization script. Blue and red spheres represent varicosities. The script's GUI is shown in the bottom right.

License. The main script is able to read the ASC file format from Neurolucida (MicroBrightField, Inc.), the most commonly used system for single neuron reconstruction, and parse the following structures: cell body contour, axonal trees, dendritic trees, spines and varicosities. The script offers several options for rendering each of these structures: levels of detail for each structure, type of cell body representation, level of interpolation in trees for smoother representation, which types of structures should be rendered, etc. All trees (axonal and dendritic) are created using an algorithm which produces a single mesh for each tree. The tree mesh is built in sequence, with each new raw 3D point being used to pull and grow the mesh towards the new point. Interpolation, if selected, uses cubic Bezier curves to smooth tree curvatures. The construction of the models from the raw data is very fast and trees take only a few seconds to be built and rendered, even if they are made up of several thousand 3D points. Varicosities are slower to render and a neuron with a few thousand varicosities can take several minutes to be reconstructed. The graphical user interface (GUI) for the main script and a snapshot of a reconstructed neuron visualized with the script, are shown in Figure 1.

In addition to the main script, a collection of python functions were created to perform measurements and calculations on the parsed neuron data. These functions are accessible through an interactive python command window and they allow, among other things, calculation of varicosities densities, fiber lengths and inter-spine distances. The scripts can easily be extended to incorporate new functions.

